# Ventriculostomy-associated infection (VAI) in patients with acute brain injury—a retrospective study

**DOI:** 10.1007/s00701-024-06018-w

**Published:** 2024-03-11

**Authors:** Pernille Nielsen, Markus Harboe Olsen, Rasmus Stanley Willer-Hansen, John Hauerberg, Helle Krogh Johansen, Aase Bengaard Andersen, Jenny Dahl Knudsen, Kirsten Møller

**Affiliations:** 1 Department of Neuroanaesthesiology, Neuroscience Centre, Copenhagen University Hospital – Rigshospitalet, Copenhagen, Denmark; 2 Copenhagen Neuroanaesthesiology and Neurointensive Care Research Group (CONICA), Copenhagen, Denmark; 3 Department of Urology, Zealand University Hospital, Roskilde, Denmark; 4 Department of Neurosurgery, Neuroscience Centre, Copenhagen University Hospital – Rigshospitalet, Copenhagen, Denmark; 5 Department of Clinical Microbiology, Diagnostic Centre, Copenhagen University Hospital – Rigshospitalet, Copenhagen, Denmark; 6 Department of Clinical Medicine, Faculty of Health Sciences, University of Copenhagen, Copenhagen, Denmark; 7 Department of Infectious Diseases, Copenhagen University Hospital – Rigshospitalet, Copenhagen, Denmark

**Keywords:** Acute brain injury, Ventriculostomy associated infections, EVD infections, Central nervous system infections, Infections, Bacterial infections and mycoses, Catheter-related infections, Central nervous system infections, Cerebrospinal fluid shunts, Ventriculostomy, Drainage, Brain injuries, Cerebrovascular disorders, Postoperative complications

## Abstract

**Background:**

Ventriculostomy-associated infection (VAI) is common after external ventricular drains (EVD) insertion but is difficult to diagnose in patients with acute brain injury. Previously, we proposed a set of criteria for ruling out VAI in traumatic brain injury. This study aimed to validate these criteria. For exploratory purposes, we sought to develop and validate a score for VAI risk assessment in patients with different types of severe acute brain injury.

**Methods:**

This retrospective cohort study included adults with acute brain injury who received an EVD and in whom CSF samples were taken over a period of 57 months. As standard non-coated bolt-connected EVDs were used. The predictive performance of biomarkers was analyzed as defined previously. A multivariable regression model was performed with five variables.

**Results:**

A total of 683 patients with acute brain injury underwent EVD placement and had 1272 CSF samples; 92 (13.5%) patients were categorized as *culture-positive VAI*, 130 (19%) as *culture-negative VAI*, and 461 (67.5%) as *no VAI*. A low CSF WBC/RBC ratio (< 0.037), high CSF/plasma glucose ratio (> 0.6), and low CSF protein (< 0.5g/L) showed a positive predictive value of 0.09 (95%CI, 0.05–0.13). In the multivariable logistic regression model, days to sample (OR 1.09; 95%CI, 1.03–1.16) and CSF WBC/RBC ratio (OR 34.86; 95%CI, 3.94–683.15) were found to predict VAI.

**Conclusion:**

In patients with acute brain injury and an EVD, our proposed combined cut-off for ruling out VAI performed satisfactorily. Days to sample and CSF WBC/RBC ratio were found independent predictors for VAI in the multivariable logistic regression model.

**Supplementary Information:**

The online version contains supplementary material available at 10.1007/s00701-024-06018-w.

## Introduction

In patients with acute brain injury, such as hemorrhagic stroke and traumatic brain injury (TBI), external ventricular drains (EVDs) are frequently used to divert cerebrospinal fluid (CSF) and monitor intracranial pressure (ICP) [1]. The most common complication to EVD treatment is ventriculostomy-associated infection (VAI) [2], which frequently is due to the easier access for microorganisms to the intrathecal space. Patients with VAI are at risk of prolonged duration of hospital stay and EVD treatment as well as a need for permanent CSF drainage device [3]. Previous studies have found several risk factors associated to VAI (Supplemental material), but still the reported prevalence of VAI varies in the literature from 6 [4] to 36% [5] of all patients with an EVD; two meta-analyses have suggested prevalence’s of 11% [3] and 23% [6], respectively. This variation is probably due to the lack of standardized diagnostic criteria [7, 8].

### The diagnostic challenge

Typical clinical signs and symptoms associated with VAI are fever, headache, neck stiffness, cranial nerve deficits, seizures, and altered mental state [6, 8]. These symptoms are often difficult to distinguish from the clinical presentation in uninfected patients with acute brain injury, due to the damage or inflammation caused by the brain damage [9]. Patients that receive EVDs are typical critically ill and furthermore, often sedated and may have extracranial infection, such as ventilator-associated pneumonia, urinary tract infection or catheter-related bloodstream infection [10, 11]. All these factors render VAI difficult to diagnose accurately from clinical and paraclinical examinations. The currently most important test for diagnosing VAI therefore is the detection of microorganisms cultured from CSF, which, however, may lead to a delay of up to 10 days before a definitive answer is provided [12]. Moreover, this test does not in itself distinguish between contamination, colonization, and infection [6, 8]. Therefore, there is a need for faster and more accurate diagnostics. Previously, Willer-Hansen et al. proposed three criteria of CSF WBC/RBC ratio < 0.037, low CSF protein < 0.5g/L, and high CSF/P glucose ratio > 60%, which, when combined, appear to have a positive predictive value (PPV) at 0 (95% confidence interval (CI), 0.0–0.14) for VAI in patients with TBI, thereby effectively ruling out VAI [13].

The aim of this study was to validate the previously proposed cut-off for ruling out VAI in a broader population of patients with acute brain injury, in whom an EVD has been inserted [13]. Furthermore, we exploratory aimed to develop a score for estimating the risk of VAI, using a combination of readily available biomarkers to guide clinicians in their decision on whether to begin treatment.

## Methods

Medical records were retrospectively screened for patients ≥ 18 years with acute brain injury, in whom an EVD was inserted at the Department of Neurosurgery, Copenhagen University Hospital - Rigshospitalet, Copenhagen, Denmark, between 6 November 2016 and 25 July 2021. The study was approved by the Directors of the Neuroscience Centre and the Departments of Neurosurgery and Neuroanaesthesiology, respectively, at Rigshospitalet (protocol id: 20069795), and approved as a register study from Centre of Regional Development (file number: R-21047752). According to Danish law, no other approval was necessary.

Acute brain injury was defined as TBI, spontaneous intracerebral hemorrhage (ICH), or non-traumatic subarachnoid hemorrhage (SAH). Non-traumatic SAH was divided by the underlying cause, i.e., an aneurismal bleed or other causes (such as, but not restricted to, an arteriovenous malformation). Patients with pre-existing cerebral infection (e.g., meningitis, cerebral abscess) or having ventriculoperitoneal (VP) or ventriculoatrial (VA) shunts, tumors, ischemic apoplexies, and patients in whom an EVD was placed electively for diagnostic purposes, were excluded.

EVDs were inserted under sterile conditions in the operating room. As standard non-coated, bolt-connected EVDs were used. If an EVD had to be replaced the procedure was done either in the operating room or at the intensive care unit. During the admission, CSF samples were collected under sterile conditions at the discretion of the attending clinician. Samples were sent to the Department of Clinical Biochemistry for biochemical analyses and to Department of Clinical Microbiology for microscopy, culturing, and antibiotic susceptibility testings.

### Data collection

From medical records, the following was registered: age. gender, time of ictus, diagnosis, characteristics of EVD (placement, localization, replacement, and removal), neurosurgical or endovascular procedures, treatment characteristics (antibiotic treatment and treatment length), survival status, biochemical laboratory parameters (plasma and CSF), and microbiological data. CSF and plasma samples were collected on the same day.

### Terminology

**Fig. 1:**
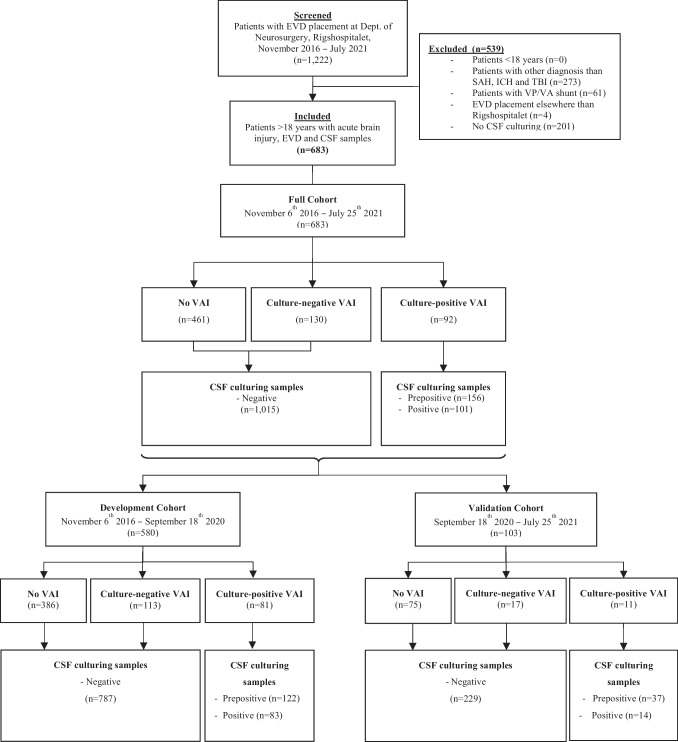
Diagram with patient classification. EVD, external ventricular drain; CSF, cerebrospinal fluid; VAI, ventriculostomy associated infections; SAH, subarachnoid hemorrhage; ICH, intracranial hemorrhage; TBI, traumatic brain injury; VP/VA shunt, ventriculoperitoneal or ventriculoatrial shunt

The patients were classified as follows (Figure 1): *culture-positive VAI* for patients treated for VAI that had a positive CSF culture, which was not attributed to contamination; *culture-negative VAI* for patients treated with antibiotics for suspected VAI but with negative CSF culture; and *no VAI* for patients never treated for VAI and who had negative CSF cultures throughout the EVD treatment period.

CSF samples were categorized as *negative*, *pre-positive*, or *positive*. A *positive* sample corresponded to the patient’s first sample that was culture-positive, while *pre-positive* was used for samples drawn before the *positive* sample in these patients (Supplemental material). *Pre-positive* samples and samples collected after a positive sample were excluded. A *negative* sample was used for samples in patients with *no VAI*, and for all samples drawn prior to antibiotic treatment targeting VAI in patients with c*ulture-negative VAI* **(**Table 2).

### Nomenclature

Willer-Hansen et al. used the term “ventriculostomy-related infection (VRI),” for the sake of clarity, we decided consistently to use the term “ventriculostomy-associated infection (VAI)” to refer to the same concept, in alignment with their publication. The same applies to other synonymous terms representing EVD-related infections.

### Outcomes

The primary outcome was the performance of the proposed cut-off for ruling out VAI by Willer-Hansen et al. [13]. We used the same definitions of pleocytosis (CSF WBC/RBC ratio > 0.037), hypoglycorrhachia (CSF/plasma glucose ratio < 0.6 [14]), and increased CSF protein (> 0.5g/L) and calculated the sensitivity, specificity, positive predictive-, and negative predictive values. For this outcome, CSF samples were classified as either *positive* or *negative*, thereby excluding the pre-positive samples.

### Data handling and statistical analyses

Data were stored in REDCap (REDCap version 12.0.3 Vanderbilt University, Tennessee, United States of America). Statistical analyses were carried out using R (R version 4.2.2, R Core Team 2022, Vienna, Austria). Continuous variables were tested for normality using Shapiro-Wilk test for normality. Normally distributed data are presented using mean and standard deviations (SD), while non-normally distributed data are presented using the median and interquartile range or 1st or 3rd quartile. Categorical variables are presented as counts and percentages. *p* < 0.05 was considered statistically significant.

For validation of the tree combined criteria for ruling out VAI, sensitivity, specificity, positive predictive value (PPV), and negative predictive value (NPV) were calculated for both individual and combined criteria.

A multivariable logistic regression was performed to analyze the association between positive and negative CSF culture and five variables with potential association to VAI [6, 15–23]: number of days with EVD, CSF WBC/RBC ratio, CSF/plasma glucose ratio, CSF neutrophil granulocyte (NG)/WBC ratio, and CSF protein (Table 5).

As an exploratory outcome, we intended to evaluate the performance of a machine learning model as derived in a development cohort and tested in a validation cohort. It has since become apparent that decisions regarding the design probably contributed to the poor performance of, and introduced limitations, to the model. In the interest of full disclosure, methods and results regarding the model are described in full in the Supplemental material.

## Results

From 6 November 2016 to 25 July 2021, we identified 683 patients with acute brain injury who were treated with an EVD and had at least one CSF sample drawn (Figure 1). Ninety-two (13.5%) patients were classified as *culture-positive VAI*, 130 (19%) patients as *culture-negative VAI*, and 461 (67.5%) patients as *no VAI* (Table 1). The number of EVD placements was highest among patients with acute brain injury in 2017, coinciding with the peak occurrence of culture-positive VAI cases (Supplemental material). The distribution between *No VAI*, *culture-negative VAI*, and *culture-positive VAI* varied between different types of acute brain injury (Supplemental material)

**Table 1:** Baseline characteristics

	Total, *N* = 683 (100%)	No VAI, *N* = 461 (67.5%)	Culture-negative VAI, *N* = 130 (19%)	Culture-positive VAI, *N* = 92 (13.5%)
Age, years*—median [Q1; Q3]*	61 [50; 71]	62 [52; 71]	59 [48; 71]	57 [47; 68]
Male sex*—N (%)*	329 (48.2)	229 (49.7)	55 (42.3)	45 (48.9)
Diagnosis*—N (%)*				
Aneurismal SAH	301 (44.1)	174 (37.7)	81 (62.3)	46 (50.0)
ICH	234 (34.3)	180 (39.0)	26 (20.0)	28 (30.4)
TBI	101 (14.8)	76 (16.5)	13 (10.0)	12 (13.0)
Non-aneurismal SAH	47 (6.9)	31 (6.7)	10 (7.7)	6 (6.5)
Surgical and endovascular procedures other than EVD placement—*N (%)*				
Craniotomy	217 (31.8)	148 (32.1)	47 (36.2)	22 (23.9)
Craniectomy	31 (4.5)	19 (4.1)	6 (4.6)	6 (6.5)
Endovascular	186 (27.2)	108 (23.4)	44 (33.8)	34 (37.0)
Reoperation ≥ 1	105 (15.4)	60 (13.0)	24 (18.5)	21 (22.8)
Surgical procedures related to EVD—*N (%)*				
EVD inserted bilaterally	89 (13.0)	58 (12.6)	11 (8.5)	20 (21.7)
EVD replacement	130 (19.0)	67 (14.5)	27 (20.8)	36 (39.1)
EVD duration, days*—median [Q1; Q3]*	16 [12; 19]	15 [11; 18]	17 [13; 20]	21 [16; 27]
Time to treatment, days—*median [Q1; Q3]*	9 [7; 13]	-	8 [6; 11]	12 [8; 17]
Treatment duration days—*median [Q1; Q3]*	6 [4; 9]	-	6 [4; 8]	7 [5; 10]
First treatment for VAI*—N (%)*				
IT gentamicin	4 (0.6)	-	0 (0.0)	4 (4.3)
IT vancomycin	219 (32.1)	-	129 (99.2)	90 (97.8)
Other antibiotics	9 (1.3)	-	2 (1.5)	7 (7.6)
Definite treatment after EVD*—N (%)*				
Removal, no further drainage needed	493 (72.2)	340 (73.8)	87 (66.9)	66 (71.7)
VP shunt inserted	183 (26.8)	117 (25.4)	41 (31.5)	25 (27.2)
Transfer to other hospital with EVD still in situ	7 (1.0)	4 (0.9)	2 (1.5)	1 (1.1)
Mortality*—N (%)*				
With EVD in situ	58 (8.5)	46 (10.0)	9 (6.9)	3 (3.3)
30-day mortality	92 (13.5)	69 (15.0)	17 (13.1)	6 (6.5)

*EVD* external ventricular drain, *ICH* intracerebral hemorrhage, *IT* intrathecal, *N* number, *SAH* subarachnoid hemorrhage, *SD* standard deviation, *TBI* traumatic brain injury, *VP* ventriculoperitoneal, *Q1* 1st quartile, *Q3* 3rd quartile

**Fig. 2:**
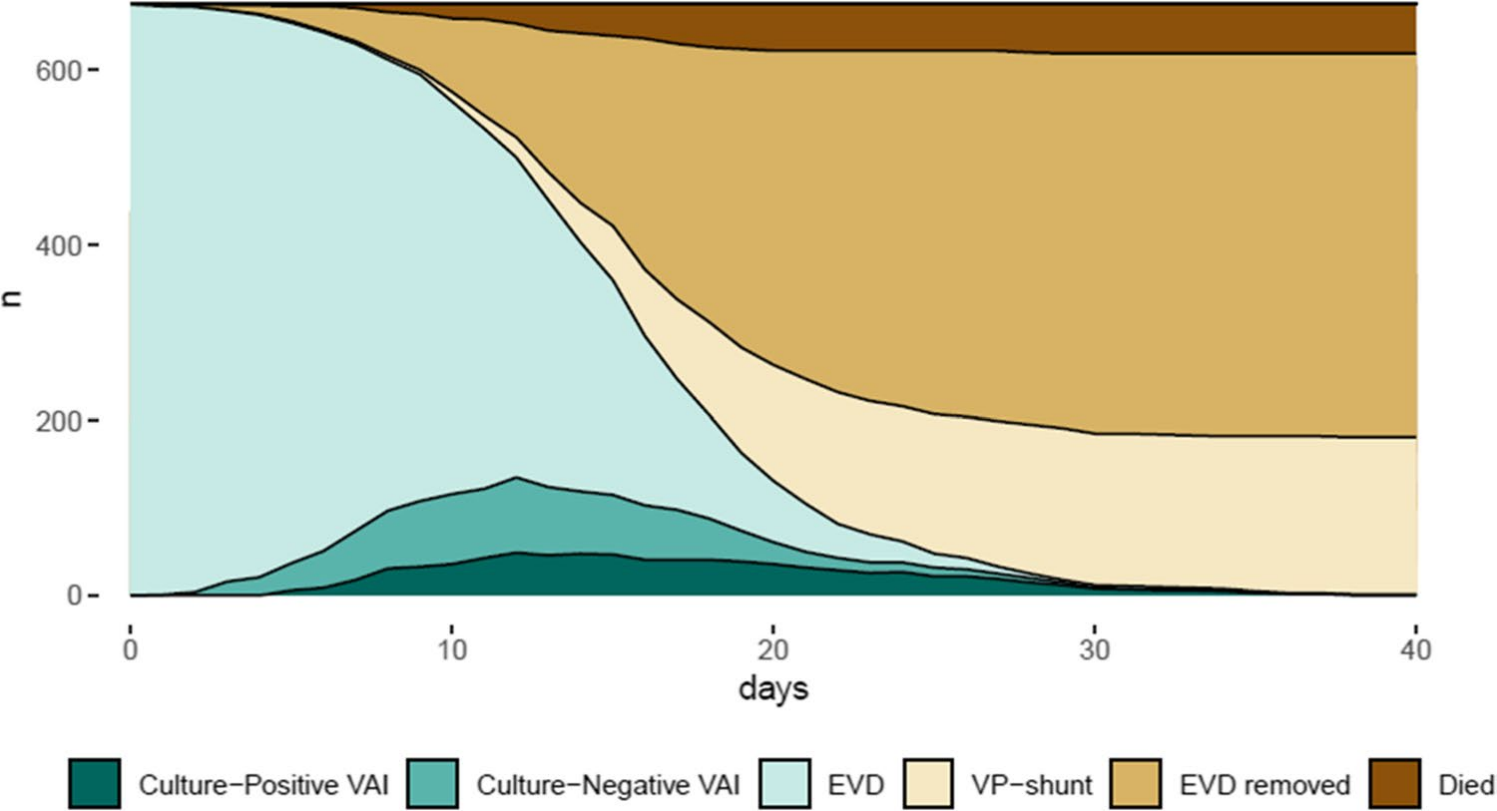
Timeline. Day 0 refers to the day of the EVD placement. EVD, all patients included in the study; EVD removed, patients who had their EVD removed; VP shunt, patients who had their EVD replaced by a VP shunt; died, patients who died; culture positive VAI, patients treated for VAI with a culture-positive sample; culture negative VAI, patients who were treated for VAI at the clinicians’ discretion, but with culture-negative samples

Patients with *culture-positive VAI* had their EVD *in situ* for longer time, more frequently had bilateral EVDs, and were more likely to experience EVD replacement than patients with *no VAI*. Intrathecal (IT) antibiotic treatment was given to almost all patients with *culture-negative VAI* or *culture-positive VAI* in the form of vancomycin 20 mg once daily. Patients with *culture-positive VAI* received treatment for a median of 7 days (Q1; Q3: 5; 10), and patients with *culture-negative VAI* for 6 days (Q1; Q3: 4; 9) days (Fig. 2, Table 1).

### CSF samples

In total, 1272 CSF samples were drawn for microbial culture and biochemical analysis (Table 2). The median number of samples per patient was 2 (Q1; Q3: 1; 3). The *positive* samples had overall higher values of CSF-WBC, CSF-WBC/RBC, CSF NG, and CSF NG/WBC than the *negative* samples, whereas the CSF/plasma glucose ratio was lower in the positive samples (Table 2). Eighty-nine CSF cultures were culture-positive, of which 89% yielded Gram-positive bacteria (most commonly *Staphylococcus epidermidis*) and 8% Gram-negative bacteria; fungi were present in 3% (Table 3).

**Table 2:** Biochemical characteristics

	Negative (*N* = 1015)	Positive (*N* = 101)	*p*-value*
Days to sample—*median [Q1; Q3]*	9 [6; 13] *(N = 1015)*	10 [7; 16] *(N = 101)*	*0.001*
CSF WBC—*median [Q1; Q3]*	72 [22; 223] *(N = 971)*	111 [24; 562] *(N = 98)*	*0.03*
CSF RBC—*median [Q1; Q3]*	11,200 [3500; 27,500] *(N = 966)*	6,400 [1300; 19,800] *(N = 97)*	*0.002*
CSF glucose—*median [Q1; Q3]*	4.3 [3.7; 5] *(N = 1005)*	4.1 [3.4; 4.8] *(N = 101)*	*0.02*
CSF/plasma glucose—*median [Q1; Q3]*	0.58 [0.49; 0.67] *(N = 871)*	0.53 [0.45; 0.64] *(N = 84)*	*0.02*
CSF protein—*median [Q1; Q3]*	0.46 [0.27; 0.75] *(N = 1009)*	0.48 [0.24; 0.81] *(N = 101)*	*0.47*
CSF neutrophil granulocytes—*median [Q1; Q3]*	25 [5; 110] *(N = 828)*	60 [10; 323] *(N = 82)*	*0.002*
CSF NG/WBC—*median [Q1; Q3]*	0.33 [0.12; 0.54] *(N = 813)*	0.40 [0.16; 0.70] *(N = 82)*	*0.007*
CSF WBC/RBC—*median [Q1; Q3]*	0.01 [0.0; 0.02] *(N = 938)*	0.02 [0.0; 0.08] *(N = 96)*	*< 0.001*
Blood CRP—*median [Q1; Q3]*	18 [7; 45] *(N = 906)*	17 [8; 47] *(N = 83)*	*0.99*

*CRP* C-reactive protein, *CSF* cerebrospinal fluid, *N* number, *NG* neutrophil granulocytes, *RBC* red blood cell count, *Q1* first quartile, *Q3* third quartile, *WBC* white blood cell count

*p-values are found using Mann-Whitney *U*-test

**Table 3:** Microbiological cultures

	Bacteria	Number of occurrences	Proportions
			(%)
G+	*Staphylococcus epidermidis*	30	33.7
	Other *coagulase-negative Staphylococci*	25	28.1
	*Enterococcus faecium*	8	9.0
	*Corynebacterium species*	4	4.5
	Non-hemolytic *Steptococcus mitis*	3	3.4
	Non-hemolytic *Streptococcus sanguinis gr.*	2	2.2
	*Staphylococcus aureus*	1	1.1
	*Micrococcus luteus*	1	1.1
	*Micrococcus species*	1	1.1
	*Aerococcus viridans*	1	1.1
	*Bacillus species*	1	1.1
	*Coryneform rods*	1	1.1
	*Enterococcus faecalis*	1	1.1
G−	*Enterobacter cloacae*	3	3.4
	*Acinetobacter baumanii*	1	1.1
	*Klebsiella oxytoca*	1	1.1
	*Pantoea agglomerans*	1	1.1
	*Pseudomonas putida*	1	1.1
Fungi	*Candida tropicalis*	2	2.2
	*Candida albicans*	1	1.1
Total		89	100

*G−* Gram-negative, *G+* Gram-positive

### Ruling out VAI

For ruling out VAI, the combined cut-off suggested by Willer et al. [13], 9% of the patients had a *positive* sample, with a low CSF WBC/RBC ratio (< 0.037), high CSF/plasma glucose ratio (> 0.6), and low CSF protein (< 0.5g/L), whereas 91% had a *negative* sample (PPV 0.09; 95%CI, 0.05–0.13) (Table 4).

**Table 4:** Rule out VAI 2016–2021

Variables	Sensitivity (95%CI)	Specificity (95%CI)	PPV (95%CI)	NPV (95%CI)
CSF WBC/RBC ratio *(n = 1034)*	0.36 (0.27–0.47)	0.87 (0.85–0.89)	0.22 (0.16–0.3)	0.93 (0.91–0.95)
CSF/plasma glucose ratio *(n = 955)*	0.64 (0.53–0.74)	0.45 (0.41–0.48)	0.1 (0.08–0.13)	0.93 (0.90–0.95)
CSF protein *(n = 1110)*	0.47 (0.37–0.57)	0.56 (0.53–0.59)	0.1 (0.07–0.12)	0.91 (0.89–0.93)
High CSF WBC/RBC ratio and high CSF protein and low glucose ratio* *(n = 893)*	0.25 (0.16–0.36)	0.96 (0.94–0.97)	0.38 (0.25–0.53)	0.93 (0.91–0.95)
Low CSF WBC/RBC ratio and low CSF protein and high glucose ratio** *(n = 893)*	0.25 (0.16–0.36)	0.74 (0.71–0.77)	0.09 (0.05–0.13)	0.91 (0.88–0.93)

*CSF* cerebrospinal fluid, *CSF WBC/RBC ratio* white blood cell count/red blood cell count ratio, *NPV* negative predictive value, *PPV* positive predictive value

*WBC/RBC-ratio > 0.037, CSF protein > 0.5 g/L, CSF/plasma glucose ratio < 60%

**WBC/RBC-ratio < 0.037, CSF protein < 0.5 g/L, CSF/plasma glucose ratio > 60%

### Multivariable logistic regression

The multiple logistic regression analysis showed a significant increase in the odds of having a positive CSF culture associated with a higher number of days to sample (OR, 1.09; 95%CI, 1.03–1.16) and increased CSF WBC/RBC ratio (OR 34.86, 95%CI 3.94–683.15). Additionally, the CSF/plasma glucose ratio (OR, 0.73; 95%CI 0.09–5.73), CSF protein (OR, 1.21; 95%CI 0.71–1.88), and CSF NG/WBC ratio (OR 2.74, 95%CI 0.78–9.57) were not found as significant predictors for a positive CSF culture (Table 5).

**Table 5:** Multiple logistic regression

Variables	OR (95%CI)	*p-*value
Days to sample	1.09 (1.03–1.16)	0.005
CSF WBC/RBC ratio	34.86 (3.94–683.15)	0.008
CSF/plasma glucose ratio	0.73 (0.09–5.73)	0.767
CSF protein	1.21 (0.71–1.88)	0.429
CSF NG/WBC ratio	2.74 (0.78–9.57)	0.114

*CSF* cerebrospinal fluid, *CSF NG/WBC ratio* CSF neutrophil granulocytes/white blood cell ratio, *CSF WBC/RBC ratio* white blood cell count/red blood cell count ratio, *OR* odds ratio, *95%CI* 95% confidence interval

### VAI prediction score

The VAI prediction score was derived in the *development cohort*, in which it showed moderate accuracy (area under the curve (AUC) 0.69; 95%CI, 0.62–0.77) (Supplemental material). In the *validation cohort*, it performed no better than chance (AUC 0.46; 95%CI, 0.30–0.79). Similarly, Youden’s threshold showed moderate accuracy (AUC 0.69; 95%CI, 0.61–0.77) in the *development cohort*, and low accuracy in the *validation cohort* (AUC 0.55; 95%CI, 0.30–0.79).

## Discussion

In this retrospective study of 683 patients with acute brain injury, we found a PPV for having a *positive* sample of 9%, when using the combined cut-off for ruling out VAI identified by Willer et al. [13].

In our cohort, patients with *culture-positive VAI* had their EVD for longer time than patients with *no VAI*. Although prolonged EVD treatment is an accepted risk factor for VAI [8, 15, 24–26], it may also be a consequence of the time waiting for antibiotic treatment effect and repeatedly negative CSF cultures in patients needing replacement of the EVD with a permanent internal shunt. Also, *culture-positive VAI* patients were more likely to undergo EVD replacement, compared to *no VAI* patients, which could be attributed to potential dysfunction or leak from the EVD, or a decision to treat VAI with intraventricular antibiotics. Our cohort resembled those in previous reports with regard to culture-positive rate and the biochemical patterns of neutrophil pleocytosis and hypoglycorrhachia [6, 13, 15, 17, 21, 27]. Unfortunately, we were unable to test the added diagnostic benefit of CSF lactate and Gram staining in our cohort as CSF lactate is not measured routinely in our department, and the CSF Gram stain was difficult to retrieve due to the retrospective design.

Several preventive measures have been explored in prior studies to reduce the incidence of VAI. These include utilization of EVD catheters coated with antimicrobial materials [28, 29], prophylactic administration of antibiotics [30], minimizing the frequency of CSF samples [31], and fixation to the skin with either tunneled or bolted EVDs [32]. Notwithstanding these efforts, in patients in whom the clinician suspects VAI, distinguishing between the inflammatory response arising from acute brain injury and infection remains challenging [9]. Koopman et al. investigated the time course of simple CSF variables for 20 days in patients with aSAH. The authors reported a significant increase in CSF leucocyte count six days post-ictus, accompanied by a decrease in RBC count; moreover, CSF protein stayed elevated 20 days after ictus [33]. Together, these findings emphasize the need for better multivariable diagnostic tools. Muñoz-Gómez et al. tested a combination of CSF lactate > 6 nmol/L, CSF pleocytosis (> 50 WBC/mm^3^), a positive CSF Gram stain, and a positive CSF culture of neuropathogens (with the same morphology) [34], although this did not yield to any false-positive results in their validation; only 22 patients were studied, of whom only one had VAI. Consequently, while this combination might be of interest, it does not avoid the time-consuming process of CSF culturing. Boeer et al. proposed a predictive model for positive CSF cultures, using a classification and regression trees analysis. The combination of CSF IL-6, blood leukocyte count, and plasma C-reactive protein, yielded an area under the receiver-operating characteristic curve of 0.89 (no CI), with a high sensitivity, specificity, and NPV [35]. Others have also tested diagnostic criteria for postoperative meningitis, but these studies were not limited to patients with EVDs and acute brain injury [18, 36].

### Predictors for VAI

According to the multivariable logistic regression, days to sample and CSF WBC/RBC ratio were independent predictors for a positive CSF culture, whereas CSF/plasma glucose ratio, CSF protein, and CSF NG/WBC ratio were not associated with a positive CSF culture. These findings align with the 2017 recommendations of the Infectious Diseases Society of America, underscoring the limitations of relying solely on abnormalities in CSF cell count, glucose, and protein as reliable indicators for the presence of infection [37]. However, several studies have found associations between these markers and VAI in univariate analyses [6, 15–17, 19–21, 23].

### Diagnostic challenges

As touched upon above, the clinical diagnosis of intracranial infection, and in particular VAI, is notoriously difficult in patients with acute brain injury because of pre-existing sedation or impaired consciousness, intracranial inflammation, and variable levels of intracranial bleeding. CSF culture is a slow procedure and may also be unreliable because the frequent use of systemic antibiotics may produce false-negative results on one side, whereas contamination and colonization may yield false-positive results on the other side [8]. The diagnosis has conventionally been left to the treating clinician based on the presence or absence of positive CSF cultures, CSF biochemical analyses, an assessment of the clinical condition, and an evaluation of the magnitude of attributable risk that infection represents for this particular patient. Thus, a recent meta-analysis found no association between VAI and increased mortality or worsened neurological outcome [3], but the author recommended the results be interpreted with caution, due to the heterogeneity of the included studies. Finally, the decision to initiate treatment implies consideration of whether continuous CSF diversion will still be needed on the short or long run.

Not surprisingly, this complex decision-making process is associated with a large variation in treatment between clinicians, hospitals, and countries [8, 38–40], potentially leading to both under- and overtreatment. The exploratory VAI prediction score was conducted to simplify this process, enable early prescription of adequate antibiotics in patients with VAI, and avoid or minimize inadequate treatment in those without VAI. This attempt proved largely unsuccessful, which points to the need for further, more in-depth analysis of clinical and biochemical features in large cohorts.

## Strengths and limitations

The strength of this study is the large sample size, considering that the cohort consisted exclusively of patients with acute brain injury. There are, however, also several limitations, most notably the retrospective design with weaknesses regarding available and standardized data. The uneven distribution between the development and validation cohort was an unfortunate choice on our part. The quantitative information did not include basic clinical information such as the level of consciousness, body temperature, systemic antibiotic treatment, or clinical signs of VAI such as neck stiffness, which could impact the study’s reliability. Notably, the prevalence of complications such as infections may be severely mis-estimated by retrospective data sampling. The statistical analyses did not adjust for covariance in the patient samples. The criteria suggested by Willer-Hansen et al. for ruling out VAI [13] were developed for TBI patients but were tested in a more diverse cohort in the present study. This study originates from the same neurointensive care unit, and some patients featured in both studies. Finally, the study protocol was not pre-published.

## Conclusion

In this study of 683 EVD-treated patients with acute brain injury, a previously suggested cut-off for ruling out VAI, consisting of a low CSF WBC/RBC ratio, a high CSF/plasma glucose ratio, and a low CSF protein, performed satisfactorily. In the multivariable logistic regression model, a longer duration from insertion to sampling and CSF WBC/RBC ratio were found independent predictors for a positive CSF culture.

## Supplementary information

ESM 1: Supplemental material (DOCX 513 kb)

## Abbreviations

AUC: Area under the curve
CSF: Cerebrospinal fluid
EVD: External ventricular drain
ICP: Intracranial pressure
IT: Intrathecal
mg: Milligrams
NG: Neutrophil granulocytes
NPV: Negative predictive value
PPV: Positive predictive value
RBC: Red blood cells
SAH: Subarachnoid hemorrhage
Sd: Standard deviation
VA shunt: Ventriculoatrial shunt
VAI: Ventriculostomy associated infections
VP shunt: Ventriculoperitoneal shunt
VRI: Ventriculostomy related infections
TBI: Traumatic brain injury
WBC: White blood cells
95%CI: 95% confidence interval
