# Supplementary material for: Ventriculostomy-associated infection (VAI) in patients with acute brain injury—a retrospective study
Source: Acta Neurochir (Wien). 2024 Mar 11;166(1):128. doi: 10.1007/s00701-024-06018-w (PMC10925569; doi:10.1007/s00701-024-06018-w)
Supplement: Supplementary file 1 — Supplemental material (DOCX 513 kb) [file 701_2024_6018_MOESM1_ESM.docx]

**Supplementary material**

Ventriculostomy Associated Infection (VAI) in Patients with Acute Brain Injury – A Retrospective Study

Content

[Supplementary table S1: Baseline Characteristics in different cohorts 3](#_Toc159486581)

[Supplementary table S2: Biomarkers 5](#_Toc159486582)

[Supplementary table S4: Confusion matrix for Ruling out VAI 7](#_Toc159486583)

[Supplementary figure S1: Timelines 8](#_Toc159486584)

[Supplementary figure S2: VAI prediction score, using Machine Learning for treshold 9](#_Toc159486585)

[Supplementary figure S3: VAI prediction score, using Youden’s treshold 10](#_Toc159486586)

[Supplementary figure S4: Biomarkers 11](#_Toc159486587)

[Supplementary figure S5: Stratification of VAI classification over years 12](#_Toc159486588)

[Supplementary material, figure legends 13](#_Toc159486589)

[Description: VAI prediction score 15](#_Toc159486590)

[References 17](#_Toc159486591)

# Supplementary table S1: Baseline Characteristics in different cohorts

| **Development cohort:**  **2016-2020** | **Total** | | **No VAI** | | **Culture-Negative VAI** | | **Culture-Positive VAI** | | **P value*** |
| --- | --- | --- | --- | --- | --- | --- | --- | --- | --- |
| **Validation cohort:**  **2020-2021** | Development | Validation | Development | Validation | Development | Validation | Development | Validation |  |
|  | N=580 | N=103 | N=386 | N=75 | N=113 | N=17 | N=81 | N=11 |  |
|  | (100%) | (100%) | (66.5%) | (72.8%) | (19.5%) | (16.5%) | (14.0%) | (10.7%) |  |
| **Age, years *–*** *median [Q1;Q3]* | 61 [50;70] | 58 [51;71] | 62 [50;71] | 63 [54;73] | 60 [48;71] | 54 [50;65] | 59 [48;68] | 49 [44; 55] | *NS* |
| **Male sex -** *N (%)* | 278 (47.9) | 51 (49.5) | 193 (50.0) | 36 (48.0) | 44 (38.9) | 11 (64.7) | 41 (50.6) | 4 (36.4) | *NS* |
| **Diagnosis –** *N (%)* | | | | | | | | | |
| -          Aneurismal SAH | 252 (43.4) | 49 (47.6) | 142 (36.8) | 32 (42.7) | 71 (62.8) | 10 (58.8) | 39 (48.1) | 7 (63.6) | *<0.001* |
| -          ICH | 198 (34.1) | 36 (35.0) | 150 (38.9) | 30 (40.0) | 21 (18.6) | 5 (29.4) | 27 (33.3) | 1 (9.1) |  |
| -          Non-aneurismal SAH | 37 (6.4) | 10 (9.7) | 23 (6.0) | 8 (10.7) | 9 (8.0) | 1 (5.9) | 5 (6.2) | 1 (9.1) |  |
| -          TBI | 93 (16.0) | 8 (7.8) | 71 (18.4) | 5 (6.7) | 12 (10.6) | 1 (5.9) | 10 (12.3) | 2 (18.2) |  |
| **Surgical and endovascular procedures - N (%)** | | | | | | | | | |
| -          Craniotomy | 183 (31.6) | 34 (33.0) | 122 (31.6) | 26 (34.7) | 41 (36.3) | 6 (35.3) | 20 (24.7) | 2 (18.2) | *0.009* |
| -          Craniectomy | 29 (5.0) | 2 (1.9) | 19 (4.9) | 0 (0.0) | 6 (5.3) | 0 (0.0) | 4 (4.9) | 2 (18.2) |  |
| -          Endovascular | 156 (26.9) | 30 (29.1) | 89 (23.1) | 19 (25.3) | 38 (33.6) | 6 (35.3) | 29 (35.8) | 5 (45.5) |  |
| -          Reoperation $\geq$1 | 88 (15.2) | 17 (16.5) | 48 (12.5) | 12 (16.0) | 22 (19.4) | 2 (11.8) | 18 (22.2) | 3 (27.3) | *0.033* |
| **Surgical procedures related to EVD** *– N (%)* | | | | | | | | | |
| -  EVD inserted bilaterally | 76 (13.1) | 13 (12.6) | 47 (12.2) | 11 (14.7) | 10 (8.8) | 1 (5.9) | 19 (23.5) | 1 (9.1) | *0.013^**^* |
| -  EVD replacement | 113 (19.5) | 17 (16.5) | 59 (15.3) | 8 (10.7) | 21 (18.6) | 6 (35.3) | 33 (40.7) | 3 (27.3) | *<0.001^***^* |

| **Development cohort:**  **2016-2020** | **Total** | | **No VAI** | | **Culture-Negative VAI** | | **Culture-Positive VAI** | | ***P value**** |
| --- | --- | --- | --- | --- | --- | --- | --- | --- | --- |
| **Validation cohort:**  **2020-2021** | Development | Validation | Development | Validation | Development | Validation | Development | Validation |  |
| **EVD duration, days *–*** *median [Q1;Q3]* | 16 [13;19] | 15 [12;19] | 15 [11;18] | 14 [10;18] | 17 [13;20] | 16 [13;20] | 21 [16;25] | 17 [16;30] |  |
| **Time to treatment, days *–*** *median [Q1;Q3]* | 9 [7;13] | 8 [6;14] | - | - | 8 [5;11] | 8 [6;13] | 12 [8;16] | 8 [7;23] | *<0.001* |
| **Treatment duration days *–*** *median [Q1;Q3]* | 6 [4;9] | 6 [4;9] | - | - | 6 [4;8] | 5 [3;7] | 7 [5;10] | 9 [5;11] | *0.005* |
| **Antibiotic treatment for VAI** *– N (%)* | | | | | | | | | |
| -          IT Gentamicin | 4 (0.7) | 0 (0.0) | - | - | 0 (0.0) | 0 (0.0) | 4 (4.9) | 0 (0.0) | *-* |
| -          IT Vancomycin | 191 (32.9) | 28 (27.2) | - | - | 112 (99.1) | 17 (100.0) | 79 (97.5) | 11 (100.0) | *-* |
| -          Other antibiotics | 8 (1.4) | 1 (1.0) | - | - | 2 (1.8) | 0 (0.0) | 6 (7.4) | 1 (9.1) | *-* |
| **Definite treatment after EVD** *– N (%)* | | | | | | | | | |
| -         Removal, no further  drainage needed | 418 (72.1) | 75 (72.8) | 286 (74.1) | 54 (72.0) | 74 (65.5) | 13 (76.5) | 58 (71.6) | 8 (72.7) | *NS* |
| -         VP shunt inserted | 155 (26.7) | 28 (27.2) | 96 (24.9) | 21 (28.0) | 37 (32.7) | 4 (23.5) | 22 (27.2) | 3 (27.3) |  |
| - Transfer to other hospital with EVD still in situ | 7 (1.2) | 0 (0.0) | 4 (1.0) | 0 (0.0) | 2 (1.8) | 0 (0.0) | 1 (1.2) | 0 (0.0) |  |
| **Mortality** *– N (%)* | | | | | | | | | |
| - With EVD in situ | 52 (9.0) | 6 (5.8) | 40 (10.4) | 6 (8.0) | 9 (8.0) | 0 (0.0) | 3 (3.7) | 0 (0.0) | *NS* |
| - 30days mortality | 80 (13.8) | 12 (11.7) | 58 (15.0) | 11 (14.7) | 16 (14.2) | 1 (5.9) | 6 (7.4) | 0 (0.0) | *NS* |

# Supplementary table S2: Biomarkers

|  | **Negative**  **(N=1,015)** | **Pre-positive**  **(N=156)** | **Positive**  **(N=101)** |
| --- | --- | --- | --- |
| **Days to sample** –  median [Q1; Q3] | 9 [6;13]  (N=1,015) | 9 [6;14]  (N=156) | 10 [7;16]  (N=101) |
| **CSF WBC** –  median [Q1; Q3] | 72 [22;223]  (N=971) | 70 [18;177]  (N=148) | 111 [24;562]  (N=98) |
| **CSF RBC** –  median [Q1; Q3] | 11,200  [3,500;27,500]  (N=966) | 7,600  [2,900;21,000]  (N=147) | 6,400  [1,300;19,800]  (N=97) |
| **CSF Glucose** –  median [Q1; Q3] | 4.3 [3.7;5]  (N=1,005) | 4.5 [3.8;5.6]  (N=154) | 4.1 [3.4;4.8]  (N=101) |
| **CSF / Plasma Glucose** –  median [Q1; Q3] | 0.58 [0.49;0.67]  (N=871) | 0.59 [0.5;0.73]  (N=126) | 0.53 [0.45;0.64]  (N=84) |
| **CSF Protein** –  median [Q1; Q3] | 0.46 [0.27;0.75]  (N=1,009) | 0.41 [0.17;0.74]  (N=154) | 0.48 [0.24;0.81]  (N=101) |
| **CSF Neutrophil Granulocytes** –  median [Q1; Q3] | 25 [5; 110]  (N=828) | 31 [8;92]  (N=122) | 60 [10;323]  (N=82) |
| **CSF NG / WBC** –  median [Q1; Q3] | 0.33 [0.12;0.54]  (N=813) | 0.29 [0.18; 0.52]  (N=120) | 0.40 [0.16;0.70]  (N=82) |
| **CSF WBC / RBC** –  median [Q1; Q3] | 0.01 [0.0;0.02]  (N=938) | 0.01 [0.0;0.02]  (N=142) | 0.02 [0.0;0.08]  (N=96) |
| **Blood CRP** –  median [Q1; Q3] | 18 [7;45]  (N=906) | 26 [10;62]  (N=135) | 17 [8;47]  (N=83) |
| *CRP: c-reactive protein; CSF: cerebrospinal fluid; N: number; NG: neutrophil granulocytes;*  *Q1: first quartile; Q3: third quartile; RBC: red blood cell count; WBC: white blood cell count.* | | | |

**Supplementary table S3 – Risk factors associated with VAI**

*CSF: cerebrospinal fluid; EVD: external ventricular drain; ICH: intracerebral hemorrhage; spp: species.*

| **Author** | **Study design** | **Number of patients/No of patients with infection (%)** | **VAI diagnostic criteria** | **Risk factor for infection** |
| --- | --- | --- | --- | --- |
| Dorresteijn et al.[6] (2022) | Prospective, single center (2014-2017) | 103/15 (14.5%) | Infectious Disease Society of America, 2017.   - single or multiple positive CSF cultures   with   - CSF pleocytosis and/or - hypoglycorrhachia   or   - an increasing cell count   and   - clinical symptoms suspicious for ventriculitis or meningitis | 1. Length of CSF drainage |
| Hoefnagel et al.[10] (2008) | Retrospective, single center (1993-2005) | 228/53 (23.2%) | A positive CSF culture on the day that the CSF sample was obtained | 1. Frequent CSF sampling 2. Length of EVD drainage (>11days) |
| Khalaveh et al.[11] (2021) | Retrospective, single center (2008-2019) | 369/32 (8.1%)   - 11 (2.8%) colonization - 21 (5.3%) ventriculitis | Colonisation:   - Multiple positive CSF cultures (coagulase negative Staphylococci or Cutibacteria with time to positivity within 15h)   Ventriculitis   - colonization   with   - increased C-reactive protein   or   - single or multiple CSF cultures (Enterobacter cloacae, S. Aureus, Serratia spp., Streptococcus spp., enterococcus faecalis) | 1. Multiple EVD’s |
| Sweid et al.[19] (2020) | Retrospective, single center (2012-2018) | 389/12 (3.1%) | >24hours after EVD placement   - Fever (38°C for 30minutes or 38.3°C)   and CSF   - leucocytosis >10cells/mm^3^ (500RBC:1WBC) - increased protein (>50mg/dl) - decreased glucose (<50% serum glucose) - positive CSF culture | 1. EVD replacement 2. Bilateral EVDs 3. EVD duration 4. CSF leak 5. CSF output volume |
| Walek et al.[21] (2022) | Retrospective, single center  (2015-2019) | 409/9 (2.2%) | Centre for Disease Control NHSN definition meningitis/ventriculitis | 1. Prior neurosurgery 2. Placement in Neurocritical care unit 3. CSF leak |
| Zhu et al.[24] (2021) | Retrospective, single center  (2012-2017) | 248/36 (12.7%) | Centre for Disease Control NHSN definition meningitis/ventriculitis   - organisms cultured from CSF   or   - at least one sign or symptom of ventriculitis   and for CSF   - increased WBC (>300*10^9^/L with higher apocyte ratio than monocyte ratio) - elevated protein - decreased glucose | 1. CSF sampling 2. ICU stay >5 days 3. CSF drainage (>7 days) 4. ICH |

# Supplementary table S4: Confusion matrix for Ruling out VAI

**Criteria:** CSF WBC/RBC ratio <0.037 and CSF plasma/glucose ratio >60% and CSF protein

**Sample group**

|  | **Negative samples** | **Positive samples** | **Total** |
| --- | --- | --- | --- |
| **Criteria met** | 213 | 20 | 233 |
| **Critia not met** | 711 | 73 | 784 |
| **Total** | 924 | 93 | 1017 |

R package: *epiR 🡪 epi.tests:* Point estimates and 95% CIs:

- Apparent prevalence * 0.23 (0.20, 0.26)
- True prevalence * 0.09 (0.07, 0.11)
- Sensitivity * 0.22 (0.14, 0.31)
- Specificity * 0.77 (0.74, 0.80)
- Positive predictive value * 0.09 (0.05, 0.13)
- Negative predictive value * 0.91 (0.88, 0.93)
- Positive likelihood ratio 0.93 (0.62, 1.40)
- Negative likelihood ratio 1.02 (0.91, 1.14)
- False T+ proportion for true D- * 0.23 (0.20, 0.26)
- False T- proportion for true D+ * 0.78 (0.69, 0.86)
- False T+ proportion for T+ * 0.91 (0.87, 0.95)
- False T- proportion for T- * 0.09 (0.07, 0.12)
- Correctly classified proportion * 0.72 (0.69, 0.75)

* Exact CIs

**VAI**

|  | **No VAI** | **Culture-negative VAI** | **Culture-positive VAI** | **Total** |
| --- | --- | --- | --- | --- |
| **Criteria met** | 282 | 42 | 57 | 381 |
| **Critia not met** | 777 | 225 | 225 | 1227 |
| **Total** | 1059 | 267 | 282 | 1608 |

R package: *epiR 🡪 epi.tests: **Point estimates and 95% CIs: analysis for culture-positive VAI and no VAI.

- Apparent prevalence * 0.75 (0.72, 0.77)
- True prevalence * 0.21 (0.19, 0.23)
- Sensitivity * 0.80 (0.74, 0.84)
- Specificity * 0.27 (0.24, 0.29)
- Positive predictive value * 0.22 (0.20, 0.25)
- Negative predictive value * 0.83 (0.79, 0.87)
- Positive likelihood ratio 1.08 (1.01, 1.16)
- Negative likelihood ratio 0.77 (0.60, 0.99)
- False T+ proportion for true D- * 0.73 (0.71, 0.76)
- False T- proportion for true D+ * 0.20 (0.16, 0.26)
- False T+ proportion for T+ * 0.78 (0.75, 0.80)
- False T- proportion for T- * 0.17 (0.13, 0.21)
- Correctly classified proportion * 0.38 (0.35, 0.40)

* Exact CIs* Exact CIs

# Supplementary figure S1: Timelines

1. **Culture-positive VAI**


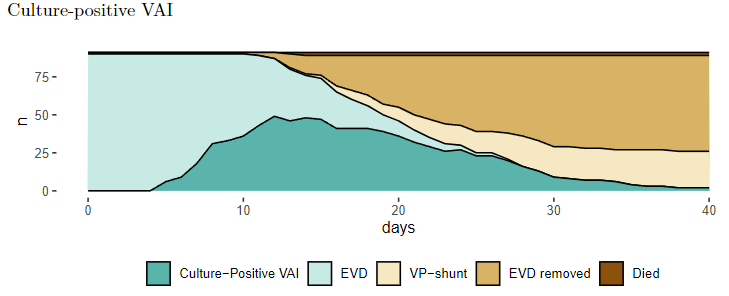


1. **Culture-negative VAI**


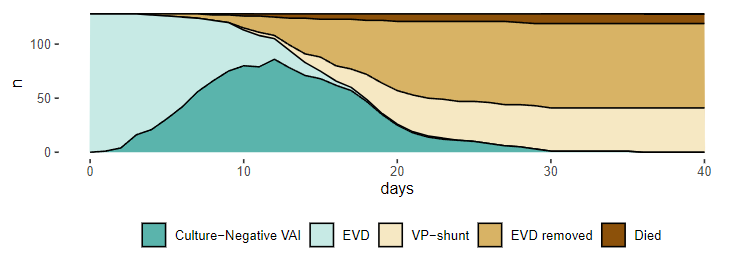


Supplementary figure S2: VAI prediction score, using Machine Learning for treshold**
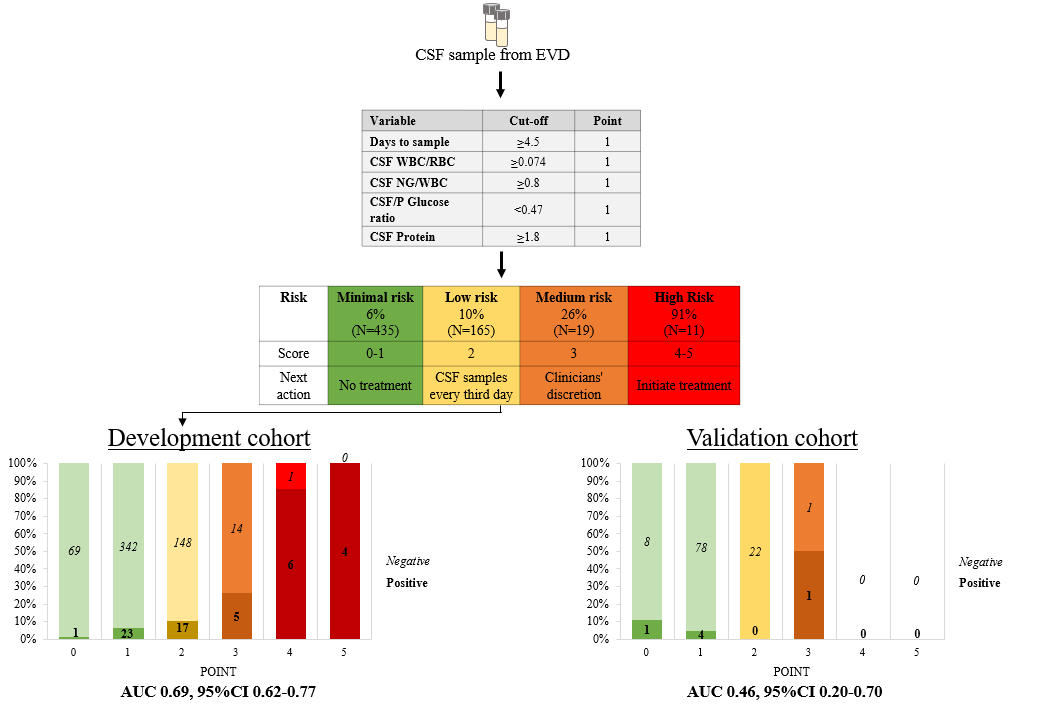
**

# Supplementary figure S3: VAI prediction score, using Youden’s treshold


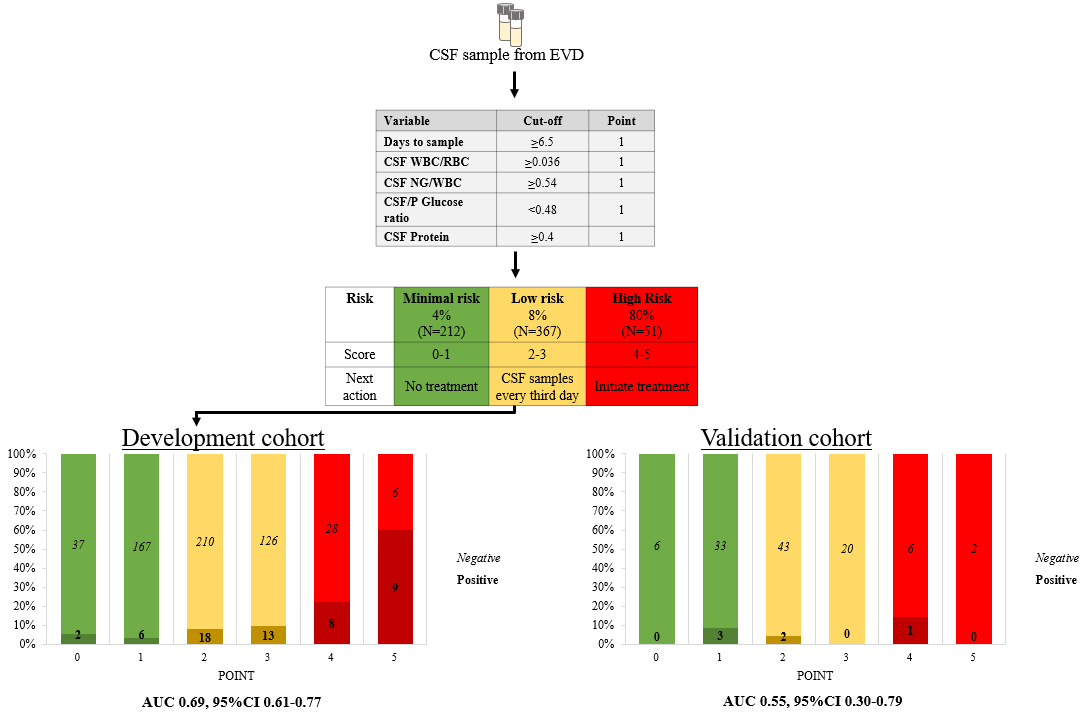


# Supplementary figure S4: Biomarkers


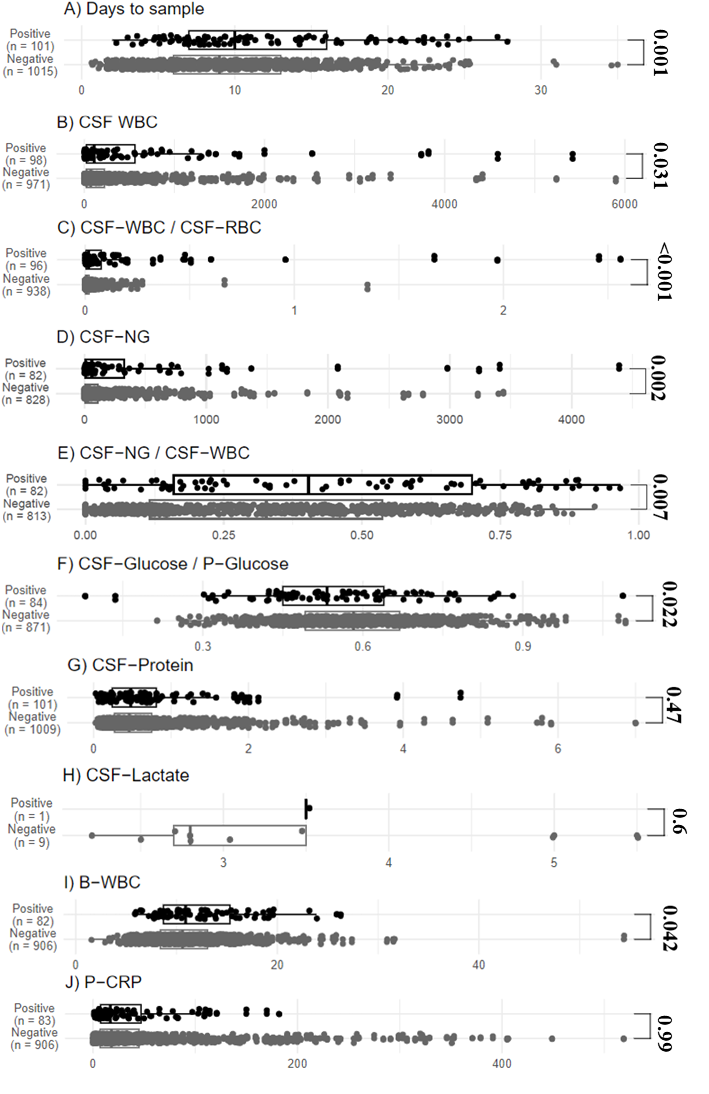


# Supplementary figure S5: Stratification of VAI classification over years


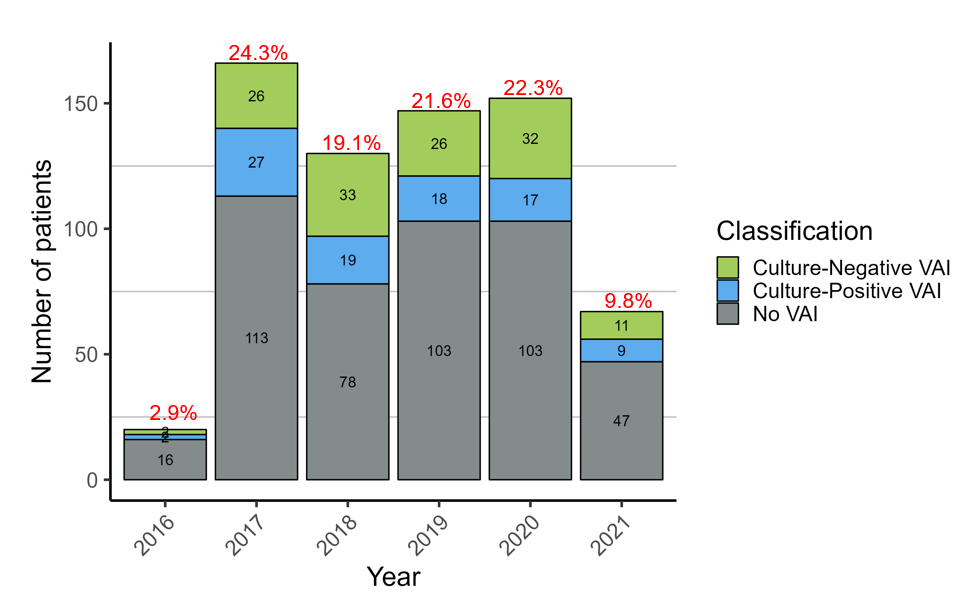


# Supplementary material, figure legends

**Supplementary figure 1 – Timelines**

See description of Figure legends, “Figure 2 – Timeline”. The supplementary figure 2, shows the timelines for a) patients with *culture-positive VAI* and b) *culture-negative VAI*.

**Supplementary figure 2 – VAI prediction score, using Machine learning**

The VAI prediction score flowchart shows how to evaluate the results from a CSF sample. The sample result will assign a point value, and the total sum results in a risk score. Increasing score increases the risk of having a subsequent positive culture. Based on the risks calculated we propose the ‘next action’. The distribution of *positive* and *negative* samples is presented in the *development cohort* and the *validation cohort*.

VAI: ventriculostomy associated infections; CSF: cerebrospinal fluid; WBC: white blood cell count; RBC: red blood cell count; NG: neutrophil granulocytes; P: Plasma;. N: number of patients

**Supplementary figure 3 – VAI prediction score, using Youden’s treshold**

The VAI prediction score flowchart shows how to evaluate the results from a CSF sample. The sample result will assign a point value, and the total sum results in a risk score. Increasing score increases the risk of having a subsequent positive culture. Based on the risks calculated we propose the ‘next action’. The distribution of *positive* and *negative* samples is presented in the *development cohort* and the *validation cohort*.

VAI: ventriculostomy associated infections; CSF: cerebrospinal fluid; WBC: white blood cell count; RBC: red blood cell count; NG: neutrophil granulocytes; P: Plasma;. N: number of patients

**Supplementary figure 4 – Biomarkers**

These figures compare the *positive* with the *negative* samples. (**A**) The number of days to sample, (**B**) CSF WBC, (**C**) CSF WBC/RBC, (**D**) CSF NG, (**E**) CSF NG/WBC and (**I**) Blood WBC is higher in the positive samples. (**F**) CSF glucose / P glucose was lower in the positive samples, while (**G**) CSF protein, (**H**) CSF lactate, and (**J**) Plasma CRP didn’t show any difference. CSF: cerebrospinal fluid; WBC: white blood cell count; RBC: red blood cell count; NG: neutrophil granulocytes; CSF-Glu: cerebrospinal fluid glucose; P-Glu: plasma glucose; B-WBC: white blood cell count in blood; P-CRP: plasma concentration of C-reactive protein; n: number of patients

**Supplementary figure 5: VAI stratified by years**

The histogram shows the stratification of the VAI classification over years. The percentage of patients with culture-positive VAI by year of all patients with culture-positive VAI is shown above the bars (red text).

2016: from 6 November 2016 to 31 December 2016. 2021; 1 January 2021 to 25 July 2021.

# Description: VAI prediction score

***Methods:***

The prediction score was generated using data from patients between 6 November 2016 and 18 September 2020 (85%; *development cohort)* – and validated using data from patients admitted in the latest period between 19 September 2020 and 25 July 2021 (15%; *validation cohort*).

The VAI prediction score was developed in the derivation cohort as a sum score based on the following five variables with a potential association to VAI[2, 3, 5, 8, 9, 13, 14, 16, 17, 22]: number of days with EVD; CSF WBC/RBC ratio; CSF/plasma glucose ratio; CSF neutrophil granulocyte (NG)/WBC ratio; and CSF protein. We constructed a multivariable logistic regression score, where each variable was given equal weight. Optimal cut-off values were investigated using machine learning, making a decision tree for each variable, finding the threshold which provided maximum differentiation among the sample groups[4, 23]. To provide a measure of risk assessment, a risk score was calculated based on these thresholds, and whether the observed values met or exceeded the defined thresholds. The total score was intended to reflect the risk of VAI, with higher scores associated with higher risk. Subsequently, we tested the score in the validation cohort by calculating the area under the receiver-operating characteristic curve; as previously suggested, we interpreted the result as chance (~0.5), low accuracy (0.5-0.7), moderate accuracy (0.7-0.9), and high accuracy (> 0.9), respectively[20]. Finally, for exploratory purposes, the VAI prediction score was also evaluated using Youden’s index analysis for finding other thresholds. Youden’s threshold is defined as the value at which sensitivity and specificity of the given marker are weighted equally.

***Results****:*

VAI prediction score

The VAI prediction score was derived in the *development cohort*, in which it showed moderate accuracy (area under the curve (AUC) 0.69, 95%CI: 0.62-0.77) (**Figure 3**). In the *validation cohort*, it performed no better than chance (AUC 0.46, 95%CI: 0.30-0.79). Similarly, Youden’s threshold showed moderate accuracy (AUC 0.69, 95%CI: 0.61-0.77) in the *development cohort*, and low accuracy in the *validation cohort* (AUC 0.55, 95%CI: 0.30-0.79).

***Discussion:***VAI prediction score cut-offs

The cut-offs in the VAI prediction score differed from previous proposed cut-off values. For CSF WBC/RBC ratio, the derived cut-off was 0.074, higher than the one reported by Brooks et al.[2], whereas Berger-Estilita et al. found this variable to be significantly associated with VAI, but observed a low sensitivity and specificity and therefore did not calculate predictive values[1]. Furthermore, we applied a CSF-protein cut-off higher than previous meta-analysis[5] and observational studies[8, 13]. Finally, the cut-off for CSF/plasma glucose ratio was similar to previous investigations[8, 14]. Although neutrophil pleocytosis is a widely accepted marker of infection[3, 9], CSF NG/WBC is only sparsely investigated as a marker for VAI. For exploratory purposes, data were tested for different cut-off values, including Youden’s threshold, but the suboptimal performance remained, probably because the criteria were unable to differentiate between infection/inflammation, colonization and ventriculitis[12, 15, 18], or to the diversity of the CSF samples, depending on presence of intraventricular hemorrhage and the ensuing inflammatory response[7]. Additionally, the small sample size in the *validation cohort* was associated with only six patients having *culture-positive VAI* and of these, none belonged to the category “high risk” of infection.

***Conclusion***

The VAI prediction score provided good discrimination in the development cohort, but performed poorly in the validation cohort, at least partly due to a low number of events in the latter.

# References

1. Berger-Estilita J, Passer M, Giles M, Wiegand J, Merz TM (2018) Modalities and accuracy of diagnosis of external ventricular drainage-related infections: a prospective multicentre observational cohort study. Acta Neurochir (Wien) 160:2039–2047. doi: 10.1007/s00701-018-3643-4

2. Brooks M, Duong D, Shivapathasundram G, Sheridan M (2022) Cerebrospinal fluid white cell count to red cell count ratio as a predictor of ventriculitis in patients with external ventricular drains. ANZ J Surg 92:3278–3282. doi: 10.1111/ans.17725

3. Citerio G, Signorini L, Bronco A, Vargiolu A, Rota M, Latronico N, Infezioni LIquorali Catetere Correlate Study Investigators (2015) External Ventricular and Lumbar Drain Device Infections in ICU Patients: A Prospective Multicenter Italian Study. Crit Care Med 43:1630–7. doi: 10.1097/CCM.0000000000001019

4. Deo RC (2015) Machine Learning in Medicine. Circulation 132:1920–30. doi: 10.1161/CIRCULATIONAHA.115.001593

5. Dorresteijn KRIS, Jellema K, Van De Beek D, Brouwer MC (2019) Factors and measures predicting external CSF drain-associated ventriculitis: A review and meta-analysis. Neurology 93:964–972. doi: 10.1212/WNL.0000000000008552

6. Dorresteijn KRIS, Verheul RJ, Ponjee GAE, Tewarie RN, Müller MCA, van de Beek D, Brouwer MC, Jellema K (2022) Diagnostic Accuracy of Clinical Signs and Biochemical Parameters for External Ventricular CSF Catheter-Associated Infection. Neurol Clin Pract 12:298–306. doi: 10.1212/CPJ.0000000000200059

7. Fam MD, Zeineddine HA, Eliyas JK, Stadnik A, Jesselson M, McBee N, Lane K, Cao Y, Wu M, Zhang L, Thompson RE, John S, Ziai W, Hanley DF, Awad IA (2017) CSF inflammatory response after intraventricular hemorrhage. Neurology 89:1553–1560. doi: 10.1212/WNL.0000000000004493

8. Grille P, Verga F, Biestro A (2017) Diagnosis of ventriculostomy-related infection: Is cerebrospinal fluid lactate measurement a useful tool? J Clin Neurosci 45:243–247. doi: 10.1016/j.jocn.2017.07.031

9. Hernández Ortiz OH, García García HI, Muñoz Ramírez F, Cardona Flórez JS, Gil Valencia BA, Medina Mantilla SE, Moreno Ochoa MJ, Sará Ochoa JE, Jaimes F (2018) Development of a prediction rule for diagnosing postoperative meningitis: a cross-sectional study. J Neurosurg 128:262–271. doi: 10.3171/2016.10.JNS16379

10. Hoefnagel D, Dammers R, Ter Laak-Poort MP, Avezaat CJJ (2008) Risk factors for infections related to external ventricular drainage. Acta Neurochir (Wien) 150:209–214. doi: 10.1007/s00701-007-1458-9

11. Khalaveh F, Fazel N, Mischkulnig M, Vossen MG, Reinprecht A, Dorfer C, Roessler K, Herta J (2021) Risk Factors Promoting External Ventricular Drain Infections in Adult Neurosurgical Patients at the Intensive Care Unit—A Retrospective Study. Front Neurol 12. doi: 10.3389/fneur.2021.734156

12. Koopman I, Zuithoff NPA, Rinkel GJE, Vergouwen MDI (2020) The course of cerebrospinal fluid parameters ≤ 20 days after aneurysmal subarachnoid hemorrhage. J Neurol Sci 415:116899. doi: 10.1016/j.jns.2020.116899

13. Lenski M, Biczok A, Neufischer K, Tonn J-C, Briegel J, Thon N (2019) Significance of cerebrospinal fluid inflammatory markers for diagnosing external ventricular drain-associated ventriculitis in patients with severe traumatic brain injury. Neurosurg Focus 47:E15. doi: 10.3171/2019.8.FOCUS19407

14. Lenski M, Huge V, Schmutzer M, Ueberschaer M, Briegel J, Tonn J-C, Schichor C, Thon N (2019) Inflammatory Markers in Serum and Cerebrospinal Fluid for Early Detection of External Ventricular Drain-associated Ventriculitis in Patients With Subarachnoid Hemorrhage. J Neurosurg Anesthesiol 31:227–233. doi: 10.1097/ANA.0000000000000496

15. Lozier AP, Sciacca RR, Romagnoli MF, Connolly ES (2002) Ventriculostomy-related infections: a critical review of the literature. Neurosurgery 51:170–81; discussion 181-2. doi: 10.1097/00006123-200207000-00024

16. Montes K, Jenkinson H, Habib OB, Esquenazi Y, Hasbun R (2019) Corrected white blood cell count, cell index, and validation of a clinical model for the diagnosis of health care-associated ventriculitis and meningitis in adults with intracranial hemorrhage. Clin Neurol Neurosurg 178:36–41. doi: 10.1016/j.clineuro.2019.01.012

17. Pfisterer W, Mühlbauer M, Czech T, Reinprecht A (2003) Early diagnosis of external ventricular drainage infection: results of a prospective study. J Neurol Neurosurg Psychiatry 74:929–32. doi: 10.1136/jnnp.74.7.929

18. Ramanan M, Shorr A, Lipman J (2021) Ventriculitis: Infection or Inflammation. Antibiot (Basel, Switzerland) 10. doi: 10.3390/antibiotics10101246

19. Sweid A, Weinberg JH, Abbas R, El Naamani K, Tjoumakaris S, Wamsley C, Mann EJ, Neely C, Head J, Nauheim D, Hauge J, Gooch MR, Herial N, Zarzour H, Alexander TD, Missios S, Hasan D, Chalouhi N, Harrop J, Rosenwasser RH, Jabbour P (2020) Predictors of ventriculostomy infection in a large single-center cohort. J Neurosurg 134:1218–1225. doi: 10.3171/2020.2.JNS192051

20. Swets JA (1988) Measuring the Accuracy of Diagnostic Systems. Science (80- ) 240:1285–1293. doi: 10.1126/science.3287615

21. Walek KW, Leary OP, Sastry R, Asaad WF, Walsh JM, Horoho J, Mermel LA (2022) Risk factors and outcomes associated with external ventricular drain infections. Infect Control Hosp Epidemiol 1–8. doi: 10.1017/ice.2022.23

22. Wong GK, Poon WS, Ip M (2008) Use of ventricular cerebrospinal fluid lactate measurement to diagnose cerebrospinal fluid infection in patients with intraventricular haemorrhage. J Clin Neurosci 15:654–5. doi: 10.1016/j.jocn.2007.03.011

23. Zhang Z, Zhang H, Khanal MK (2017) Development of scoring system for risk stratification in clinical medicine: a step-by-step tutorial. Ann Transl Med 5:436–436. doi: 10.21037/atm.2017.08.22

24. Zhu Y, Wen L, You W, Wang Y, Wang H, Li G, Chen Z, Yang X (2021) Influence of Ward Environments on External Ventricular Drain Infections: A Retrospective Risk Factor Analysis. Surg Infect (Larchmt) 22:211–216. doi: 10.1089/sur.2019.355
